# Investigation of the prevalence of female genital tract tuberculosis and its relation to female infertility:An observational analytical study

**Published:** 2012-11

**Authors:** Sughra Shahzad

**Affiliations:** *Department of Obstetrics and Gynecology, Social Security Hospital, Islamabad, Pakistan.*

**Keywords:** *Female genital tract*, *Tuberculosis*, *Tuberculous oophoritis*, *Cervicitis*, *Endometrial biopsy*

## Abstract

**Background: **Genital tuberculosis is a common entity in gynecological practice particularly among infertile patients. It is rare in developed countries but is an important cause of infertility in developing countries.

**Objective:** The present study has investigated the prevalence of female genital tract tuberculosis (FGT) among infertile patients, which was conducted at the Obstetrics and Gynecology Unit-I, Allied Hospital, affiliated with Punjab Medical College, Faisalabad, Pakistan.

**Materials and Methods:** 150 infertile women who were referred to infertility clinic were selected randomly and enrolled in our study. Patients were scanned for possible presence of FGT by examination and relevant investigation. We evaluated various aspects (age, symptoms, signs, and socio-economic factors) of the patients having tuberculosis.

**Results:** Very high frequency of FGT (20%) was found among infertile patients. While, a total of 25 patients out of 30 (83.33%) showed primary infertility and the remaining 5 cases (16.67%) had secondary infertility. Among secondary infertility patients, the parity ranged between 1 and 2. A total of 40% of patients (12 cases) were asymptomatic but infertile. Evidence of family history was found in 4 out of a total of 30 patients (13.3%), respectively. According to histopathological and bacteriological examination of endometrial biopsy and laparotomy, tuberculous endometritis was found in 20 out of a total of 25 (80%) cases, while tuberculous salpingitis and tuberculous oophoritis were found both in 2 (8%) of the cases, respectively. Only one case (4%) of tuberculosis cervicitis was found in the present study.

**Conclusion:** Although infertility is not a disease in classical sense, but it is an extremely important personal concern for many couples and a significant health problem for our profession. So, it is worthwhile to identify and evaluate the factors contributing to infertility.

## Introduction

Infertility is a worldwide problem. World Health Organization has defined infertility as failure to conceive despite over 12 months of regular and unprotected intercourse. Primary infertility is the term used to describe a couple that has never been able to conceive a pregnancy, after a minimum of one year of attempting to do so through unprotected intercourse (1, 2). 

“Secondary infertility is the term used to describe a couple who has previously been able to achieve another pregnancy” (3). Tubal infertility is very important factor constituting about 30-40% of cases. Tuberculosis, a chronic infectious disease, is one of the major etiological factors of female tubal infertility (4). In past decades, the incidence of tuberculosis increased due to homelessness, drug abuse and AIDS. Increase in pulmonary tuberculosis is accompanied by an increase in extrapulmonary tuberculosis. It is an infectious disease caused by *Mycobacterium tuberculosis*, which is an acid fast bacillus. Disease could be either primary that involves either, lungs (*M. tuberculosis*) or intestines (*M. bovis*). Secondary tuberculosis may involve all organs and systems of body including genital tract (5).

Female genital tract tuberculosis (FGT) as a cause of infertility is uncommon in developed countries. It is one of the commonest causes of infertility in developing countries (6, 7). It is therefore, suggested that every patient consulting for infertility in developing countries should be investigated for female genital tract tuberculosis (FGT). Predisposing factors include poverty, ill health, and immuno-suppression. In addition to infertility it gives rise to a number of sequalaes including chronic pelvic pain, ectopic pregnancy and pelvic adhesions. 

FGT involves mucosa of fallopian tube with or without involvement of uterus and ovaries. Spread is either hematogenous, lymphatic or direct spread from neighboring viscus. It is always secondary to tuberculosis elsewhere in the body usually the lungs. Frequency of involvement of genital organs is fallopian tubes 100%, endometrium 90%, ovaries 20%, cervix, vulva and vagina 1%. Sinha *et al* (1997) found myometrial involvement in FGT (8, 9).

Patients may suffer from infertility, pelvic pain, ill health, menstrual disturbances and irregular vaginal discharge. Sometimes, it results in oligomenorrhea and the treatment with antituberculosis drug therapy results in a normal menstrual cycle (10). Sometimes the patient is quite asymptomatic and seeks advice only because of infertility. At the other end of spectrum, patients have postmenopausal bleeding (11, 12). The incidence of genital tuberculosis is higher than one might imagine, based on the lack of reports in the literature, and may account for a significant amount of female infertility (13). 

As a rule, active extragenital foci of tuberculosis are rarely present when the genital lesion is discovered since the original pulmonary or extrapulmonary disease has usually been arrested. Infertility caused by genital tract tuberculosis is difficult entity regarding the treatment. In most early cases ovarian and reproductive function can be preserved, through IVF is required for fertility (5). The present study evaluates the prevalence of FGT among infertile patients. The effects of different parameters like age, symptoms, signs, and socio-economic factors, were also investigated on the onset of tuberculosis.

## Materials and methods

**Patient selection**

A total number of 150 patients at the reproductive age, referring to the Infertility Clinic of Allied Hospital, Faisalabad, Pakistan, were selected for the present study. Patients were scanned for the possible presence of FGT. The study was limited to female patients having infertility problem. All the patients were subjected to detailed history, clinical examination and relevant investigation. The present study was approved by the Ethical Review Committee (ERC), Allied Hospital, Punjab Medical Research Center (PMRC), Punjab Medical College, Faisalabad, Pakistan with the Registration number of IRB 00006912.

**Study design**

The present study was an analytical, observational and retrospective study. All patients who were admitted for indoor workup of infertility were classified as having primary and secondary infertility. Pre admission workup included hematological studies, semen analysis of husband and hormonal assay (FSH, LH, serum prolactin, mid luteal serum progesterone) of female partner.

**Evaluation of patient’s history**

Evaluation of patients was started from taking history and examination. The present study only considered the female factor of infertility. Male factor was excluded on the basis of history and husband semen analysis, performed on outdoor basis. It has been suggested in literature that age is statistically the most significant factor affecting fertility. The maximum prevalence of FGT is observed in 2^nd^ and 3^rd^ decades of life.

Occupation of patient’s husband was also recorded as there is a well known association of FGT with homelessness, poverty and poor socio-economic factors. The duration of marriage and the type of contraception was also taken as a part of history. Detailed sexual history including timing and frequency of coitus, dyspareunia, coital difficulties and the menstrual irregularities (if any) were also noted. In case of secondary infertility, the history of previous pregnancies, postabortal and puerperal sepsis, ectopic pregnancy, ovulation induction, ovarian cystectomy or appendicectomy were also recorded.

**Experimental investigations and techniques**

The patients underwent the laboratory test of blood hemoglobin (mg/dl), total leukocyte count (TLC), differential leukocyte count (DLC; polymorph, lymphocytes, eosinophils, basophils), erythrocyte sedimentation rate (ESR), mantoux test, chest X-ray for the presence/absence of tuberculosis, baseline trans-abdominal ultrasound for uterus, ovaries and pelvic organs and finally the endometrial biopsy. Laparoscopy was done to see the conditions of fallopian tubes, tubal potency, ovaries, tubo-ovarian masses etc. Hysterosalpingography was performed in the cases of tuberculosis having with the problem of infertility, tubal potency or the cases in which laparoscopy were difficult because of previous surgery and extreme obesity.

Histological examination of surgical specimen was done to see the presence of specific tuberculous granulomatous lesions. The specimen were obtained either by biopsy during laparotomy or during curettage. Two samples were sent to laboratory after biopsy. One, in 10% formalin for histopathalogical analysis and the second, in normal saline for the culture of *Bacillus tuberculosis*.

**Statistical analysis**

Quantitative results obtained from the patients were evaluated for statistical differences by one way analysis of variance (ANOVA) and statistical significance was defined at p<0.05. Then, multiple comparison test (Tukey's test) was used to compare the data of 150 patients. In addition, the paired sample *t*-test was used to compare the resulting means. 

## Results

One hundred and fifty cases of infertility were selected randomly for the evaluation of FGT that was found in 30 patients. This indicated a frequency of 20%. There is a population of 2 million females in gynecological practices in Obstetrics and Gynecology Unit of Allied Hospital, Faisalabad, Pakistan. The effects of various factors like age, parity, socio-economic setup and educational status was also considered in the present study. The age distribution ranged from 15-35 years. According to Table I, maximum frequency of disease was observed in 2^nd^ (21-30 years) and 3^rd^ (31-35 years) decade of life in 15 (50%) and 10 (33.3%) cases. About 80-85% cases were included in this age group (data not shown).

In secondary infertility, the parity ranged from 1-3. According to table I, almost all the patients (96.66%, n=29) belonged to lower socio-economic group but only one (3.33%) of the cases was seen in the higher socio-economic group. In this study, infertility was chief complaint, however, patients presented different symptoms including lower abdominal pain (n=3, 10%), menstrual disorder (n=5, 16.7%), Leukorrhea (vaginal discharge) in 3 patients (10% cases), pelvic mass (n=4, 13.3%), weight disturbances (n=1, 3.3%) and most of the patients were asymptmatic (Table II). 

The patients suffering with pelvic mass were diagnosed by laparotomy after taking the biopsy sample for histopathological examination. Two cases had past history of pulmonary tuberculosis and two had intestinal tuberculosis who had undergone laparotomy for intestinal problem few years back and had already taken antituberculosis treatment. Chest X-ray showed the evidence of primary infection in the patients who had suffered from primary pulmonary tuberculosis in past. Rest of the patients did not show any radiological evidence on chest X-ray (Table III). The ESR was evaluated in almost all the patients, with the range of 15-25 and 100-120 in the first and second hour, respectively. 

In addition, the other diagnostic aids, including past history, family history and chest X-Ray showed 4 out of 30 (13.3%) cases having positive results of FGT. ESR showed 24 out of 30 (80%) cases of FGT and mantoux test demonstrated 2 out of 10 (20%) of the FGT cases but histological examination of curettage or specimen biopsy proved to be of maximum help giving 100% results (Table III). According to table IV, the duration of infertility ranged from 2-15 years. In total 25 (83.33%) cases had primary and 5 (16.67%) had secondary infertility (Table IV).

Main stay of diagnosis was histological examination of endometrial curettage and biopsy specimen obtained during laparotomy for specific tuberculosis granulomatous lesions. Biopsies taken from appropriate sites confirmed the diagnosis on the basis of histopathological examination. Laparoscopy and diagnostic curettage was performed in all cases of primary infertility; curettage was planned for the premenstrual phase. 

Endometrial curetting was divided into two halves. One was sent for histopathological examination while the second half was cultured in Lowen Stein and Jensen medium. In our study, we had to rely mainly on the histopathology while the culture had poor yield because the patients did not submit their reports 6 weeks later and most of patients could not pay for the diagnostic tests. Results of histopathology report are given in table V.

Laparotomy was performed in those cases that showed evidence of pelvic mass, adenaxal mass on examination or abnormal pelvic scan on laparoscopy. Out of all the patients who performed laparotomy, histopathology confirmed the FGT in 4 patients (Table V).

None of the patients showed fistula or accidental injury to the bowel and bladder. Laparoscopy was performed for diagnostic purpose although it has got little diagnostic value especially to differentiate between pelvic tuberculosis and FGT unless biopsy examination has not been performed. Laparoscopy was helpful to visualize pelvic organs completely and to exclude contribution of peritoneal, tubal, ovarian and pelvic factors (data not shown). 

Laprotomy showed that Hydrosalpinx/ pyosalpinx, tubo-ovarian mass with peritubal and peri-ovarian adhesion and ruptured tubal ectopic pregnancy were seen in 3.3% (n=1) of the cases. Tuberculosis can cause the formation of mild beaded tubes or even a serious disease condition in the pelvis with dense adhesions, as seen in laparotomy (Table V).

**Table I:** Distribution of genital tuberculosis according to different age- and socio-economic groups among infertile patients

	**Age (years)**			**Socio-Economic group**	
	**15-20**	**21-30**	**31-35**	**Lower socio-economic group**	**Higher socio-economic group**
Number of cases (n=30)	5	15	10	29	01
Percentage	16.7	50.0	33.3	1	3.33

**Table II:** Signs and symptoms among infertile patients with genital tuberculosis

**Presentation**	**Number of cases**	**Percentage**
Lower abdominal pain	3	10.0
Menstrual disorders	5	16.7
Leukorrhea (vaginal discharge)	3	10.0
Weight disturbances	1	3.3
Low grade fever	1	3.3
Pelvic mass	4	13.3
Ruptured ectopic pregnancy	1	3.3
No symptoms	12	40.0

**Table III:** Results of various diagnostic aids

**Diagnostic aid**	**Number of cases**		**Percentage**
	**Total**	**Patients with positive results**	
Past history of tuberculosis	30	4	13.3
Family history of tuberculosis	30	4	13.3
ESR	30	24	80.0
Chest X-ray showing evidence of past or present tuberculosis	30	4	13.3
Mantoux test	10	2	20.0
Ultrasound scan	25	4	16.0
Histopathology of specimen on laparotomy or curettage	25	25	100.0

**Table IV:** Duration and distribution of infertility among the patients of genital tuberculosis

	**Type of infertility**		**Duration of infertility (years)**		
	**Primary**	**Secondary**	**<5**	**2-10**	**>10**
Number of cases (n=30)	25	5	12	10	8
Percentage	83.33	16.67	40.4	3	26.7

**Table V:** Main anatomic locations of genital tuberculosis and the histopathological and laparatomic investigations in the patients showing the evidence of genital tuberculosis

**Location**	**Histopathological findings**	**Total no. of cases**	**Cases with relevant disorder**	**Percentage**
Endometein	Tuberculosis endometritis	25	20	80.0
Tubes	Tuberculosis salpingitis		2	8.0
Ovaries	Tuberculosis oophoritis		2	8.0
Cervix	Tuberculosis cervicitis		1	4.0
	**Operative Findings**			
	Hydrosalpinx/pyosalpinx	5	1	3.3
Pelvis	Frozen pelvis		2	6.7
Tubo-ovarian region	Tubo ovarian mass with peritubal and periovarian adhesion		1	3.3
Tubes	Ruptured tubal ectopic pregnancy		1	3.3

## Discussion

Infertility is a health problem with very definite physiological, psychological and social implications. The stigma of infertility often leads to mental disharmony and even divorce, if the problem lies with the female partner, as in case of FGT. Although the reported incidence of genital tuberculosis in Asian and western countries varies between 0.69% in Australia and 17.4% in India, the actual incidence may be higher because a large proportion of cases go unreported due to lack of sensitive and specific investigations. Factors such as poverty, homelessness, a poorly functioning national tuberculosis program and dismantling of public health infrastructure have significantly contributed to the worsening situation (14).

Different researchers have pointed out the importance of considering the FGT in differential diagnosis of infertility particularly in younger age groups (15). Tuberculosis is commonly encountered in gynaecological practice. This disease has varying modes of presentations. In our study, most of the patients were (40%) asymptomatic, only presenting the complaint of infertility. 

According to Parasad *et al* (2012), a total of 22 (14.6%) women were diagnosed with genital tuberculosis on the basis of laboratory tests and laparoscopic/hysteroscopic findings (16). If the occurrence of infection coincides with puberty, primary infertility is caused while if infection occurs later in life, secondary infertility is seen. 

Postmenopausal bleeding is another mode of presentation of FGT that was seen in this study, as we were dealing with the population in reproductive age group. Genital tuberculosis is secondary to mycobacterial infection either in lungs or intestine (17). In some cases, the disease may be sexually transmitted from male tuberculosis epididymitis. The frequency of FGT exhibits a parallelism of pulmonary tuberculosis (18).

During the present study, different kinds of symptoms were seen in the FGT patients like lower abdominal pain (3 cases, 10%), menstrual disorders (5 cases, 16.7%), leucorrhea (3 cases, 10%), weight disturbances (1 case, 3.3%), low grade fever (1 case, 3.3%), pelvic mass (4 cases, 13.3%) and ruptured ectopic pregnancy (1 case, 3.3%). While, 12 patients (40%) did not show any symptoms. Past and family history were seen in 4 (13.3%) out of a total of 30 cases. Similar results were demonstrated by Shukla *et al* (2011) who reported that about 20% of the patients with genital TB showed a history of TB in their immediate family (19). 

According to Parasad *et al* (2012), past history of tuberculosis was present in 31.8% of patients in the form of pulmonary (42.8%), lymph node (28.5%) and bone tuberculosis (28.5%). Normal menstrual cycles were seen in 70.6% of cases while menstrual dysfunction in the form of amenorrhoea, oligomenorrhea, menorrhagia and dysmenorrhea was seen in 8%, 13.33%, 7.3% and 0.67% of women, respectively. Oligomenorrhea was found to have a significant correlation between PCR positive and negative patients (16).

The incidence of genital tract and pelvic tuberculosis has greatly diminished in Western countries along with a change in disease pattern i.e., the population suffering from it are much older and the symptomatology has changed too (20, 21) with the unusual presentation being postmenopausal bleeding or vaginal discharge. 

Though magnitude of disease is much large in developing countries but still the diagnosis of FGT is less commonly thought in gynecologic practice. Incidence of disease raises due to poverty, homelessness and malnutrition in developing countries. In addition to above mentioned fact, it is becoming an important issue in developed countries because of its association with HIV infection (22). It is well established that HIV impairs the ability to control tuberculosis infection. 

Clinical studies provide compelling evidence that HIV leads to an increased risk of developing tuberculosis shortly after HIV infection. Not only are HIV individuals at greater risk of acquiring *Mycobacterium* *tuberculosis *and developing active TB, they have an increased risk of death due to TB. Although, it has been well known over the past 25 years that HIV/*M*. *tuberculosis* co-infection is remarkably detrimental, the mechanisms by which HIV disrupts function in both established and newly forming granulomas, leading to the increased morbidity and mortality of coinfected people compared to those of people with TB alone, remain to be determined (23). 

Genital tuberculosis appears to be secondary to an initial focus elsewhere in the body, usually the lungs. However, it is not possible to find the evidence of healed or active tuberculosis as was the case in majority of our patients. It is believed that the fallopian tubes are the commonest site for genital infection constituting about 94.7% of cases of genital tuberculosis (24). 

While, the endometrium, ovaries, cervix and vagina are affected in decreasing order of frequency (25). But during the present study, 20 (80%) out of 25 patients showed tuberculosis endometritis that initiated in endometein. While, 8% (2 cases) of the subjects showed FGT in fallopian tubes. The clinical diagnosis of genital tuberculosis, especially in relation to infertility, depends upon a high index of suspicion, as this disease has varying modes of presentation (26) or patient may be completely asymptomatic. In present study, one case showed ruptured ectopic tube after the performance of laparotomy, which was proved to be because of tuberculosis salpingitis after histopathology.

Past history and family history of tuberculosis may be present in 20 and 50% cases, respectively. According to a previous study, none of the patients had past or family history of tuberculosis while the genital tuberculosis was found in seven infertile patients (27). During the present study, FGT was found in 13.3% cases showing no family history of this disease. Low incidence of a past history of pulmonary tuberculosis may be related to gastrointestinal sources of the infective organisms, illiteracy and the delay in seeking medical advice. Ovarian cancer can cause elevated levels of CA 125, but it doesn't always mean that the patient has ovarian cancer. Some women with ovarian cancer never show elevated CA 125 levels. 

Many other conditions also can cause an elevation in CA 125 concentration, including: diverticulitis, endometriosis, liver cirrhosis, normal menstruation, pregnancy, uterine fibroids, and peritoneal inflammation/irritation (peritonism). Therefore, CA 125 measurement in the patients, who are at an average risk of ovarian cancer should not be set as an mandatory diagnostic indication (28). According to certain studies, elevated levels of serum cancer antigen 125 or carbohydrate antigen 125 (CA 125) have been noted in the cases of tuberculous peritonitis (29-31). According to Shukla *et al* (2011), elevated serum CA 125 levels suggested the presence of peritoneal adhesions with cystic to firm mass in left ovary and minimal amount of free fluid in abdomen (19).

During the present study, 4 (13.3%) out of 30 cases showed the evidence of past or present tuberculosis after an X-Ray analysis of chest, which suggested a right sided pleural effusion. This evaluation was done after carefully studying the case, with past and present history along with signs, symptommatology and intra-operative findings including the parameters like chronic granulomatous lesions, endometriosis, neoplastic lesions with metastasis and Meig’s or Pseudo Meig’s syndrome (pleural effusion, ascitis and ovarian mass). 

Finally, a diagnosis of genital tuberculosis with enodmetriosis was confirmed on histopathological evaluation. Because of the importance of histopathology in the diagnosis of the disease, it is essential that there should be a closest possible consultation between the clinician and the pathologist. Endometrium should not be declared blameless in infertile patients until it has been studied completely even at ultra-structural level, which will provide answers to the unrevealed questions (14).

In our study, we investigated the infertile population and the purpose of this study was to avoid surgical treatment or laparotomy. Laparotomy was mandatory in cases that presented pelvic masses and it became important to exclude ovarian malignancy. Laparoscopy, along with hysteroscopy, is considered the gold standard for evaluating the infertility, caused by genital tuberculosis. However, any surgery–including laparoscopy-is associated with higher complication rates in patients with genital TB because of the basic underlying pathology and adhesions (32). 

According to the histopathology of the subjects on laparotomy or curettage, 25 (100%) of the patients showed positive results for FGT. While, ESR, Mantoux test and ultrasound scan showed 80% (24/30), 13.3% (2/10) and 16% (4/25 cases) cases positive for the FGT, respectively.

Operative findings on laparoscopy varied from mildly beaded tubes to very oedematous and destructed fallopian tubes with neurotizing and granulomatous structures seen in them. Laparotomy showed hydrosalpinx/pyosalpinx, Tubo ovarian mass with peritubal and peri-ovarian adhesion and ruptured tubal ectopic pregnancy in 3.3% (1 case) of the subjects, respectively. In advanced cases on laparotomy, frozen pelvis was found in 67% (n=2) of the cases and the histopathology confirmed evidence of the genital tuberculosis (32).

Treatment of infertility in FGT is the most difficult entity. At present, IVF provides the only hope. It represents useful treatment and improves the chances of fertility (33). Patients should be selected carefully, before committing them to IVF. They should meet two criteria: normal uterine cavity and functional ovaries. 

Women with genital tuberculosis appear to represent a less favorable subset within other tubal factors when treated with In Vitro Fertilization Pre-Embryo Transfer (IVF ET). Prognosis of infertility in genital tuberculosis is poor even with modern antituberculosis chemotherapy. There have been some full term pregnancy reports after chemotherapy but still the rate of term pregnancy after successful treatment of genital tuberculosis is approximately 1-2% (13, 33).

## Conclusion

Although, infertility is not a disease in classical sense, but it is an extremely important personal concern for many couples and a serious health problem. Association of pelvic infection i.e., FGT to infertility is well recognized problem because of its high frequency in our community. 

It is concluded in this study that tuberculosis is not uncommonly encountered during gynecological practice i.e., in patient-departments, wards and during surgery. Gynecologist should consider the diagnosis more frequently than is currently the case. 
